# Spatial priors affect sensory weighting in navigation and landing in Egyptian fruit bats

**DOI:** 10.1242/jeb.250551

**Published:** 2025-09-24

**Authors:** Nikita M. Finger, Keegan E. Eveland, Xiaoyan Yin, Cynthia F. Moss

**Affiliations:** ^1^ Department of Psychological and Brain Sciences, Krieger School for Arts and Sciences, Johns Hopkins University, Baltimore, MD 21218-2686, USA; ^2^ The Solomon H. Snyder Department of Neuroscience, School of Medicine, Johns Hopkins University, Baltimore, MD 21205, USA; ^3^ Department of Mechanical Engineering, Whiting School of Engineering, Johns Hopkins University, Baltimore, MD 21218, USA; ^4^ Kavli Neuroscience Discovery Institute, Johns Hopkins University, Baltimore, MD 21218, USA

**Keywords:** Memory, Attention, Egyptian fruit bats, Echolocation, Bayesian models, Inattentional blindness

## Abstract

Within the framework of Bayesian navigation models, spatial priors weigh the probability of encountering the locations of obstacles, landmarks and goals. Past work has evaluated the role of spatial priors in human and animal navigation; however, the contribution of moment-to-moment spatial attention in navigation performance in different sensory environments has not been considered. Here, we leveraged the directional aim of the Egyptian fruit bat's sonar clicks to monitor its spatial attention during navigation in light and dark environments. Priors were established by training bats to fly to a perch at a fixed location. Expectations were violated on intermittent trials by displacing the perch 15 or 30 cm. Bat flight/echolocation behavior was quantified with high-speed video and microphone array recordings. In the light, with access to vision and echolocation, bats successfully landed on the perch after it was moved 15 cm but often failed after the perch was displaced 30 cm. In the dark, using echolocation alone, bats often failed to find the perch after it was moved only 15 cm, indicating that spatial priors were weighted more heavily when vision was excluded. When bats failed to land in perch-displaced trials in the light and dark, priors guided them to direct their sonar attention to the region of the room where they expected the perch. Bat navigation failures share characteristics with inattentional blindness in humans, where priors interfere with stimulus processing. Our research demonstrates the effect of priors on spatial attention, which differentially affects sensory weighting in light and dark environments.

## Teaser

**Summary:** Leveraging the Egyptian fruit bat's vision and directional sonar flashlight reveals that memories influence spatial attention and navigation, and in different sensory environments stored information is weighed differently.

## INTRODUCTION

As humans and other animals navigate natural environments, to seek food, steer around obstacles and travel to goals, their distance and direction to objects continuously change. Such navigation behaviors in novel environments are guided largely by attending to dynamic sensory stimuli (Moss et al., 2023). In familiar environments, organisms develop expectations – spatial priors – about the environment through repeated experience (McNamara et al., 2006; Valone, 2024).

Spatial priors influence navigation across various contexts and species (McNamara and Houston, 1980; Posner, 1980; McNamara et al., 2006; de Cothi et al., 2022; Awh et al., 2024; Kessler et al., 2024; Valone, 2024). In humans, research has shown that navigation behavior is strongly influenced by learned priors, shaped by spatial memories (Kessler et al., 2024). de Cothi et al. (2022) showed that both humans and rats use spatial priors in navigation (de Cothi et al., 2022). When navigating a maze, rats and humans took efficient detours and shortcuts using a predictive map of the environment to anticipate future events, based on current and previous actions. Awh et al. (2024) showed that rats trained on multiple maze configurations continued to rely on prior experience when learning new spatial rules, providing evidence that spatial priors persist long after initial learning (Awh et al., 2024).

Bayesian models provide a mathematical framework to measure the weighting of priors with current sensory input during navigation (McNamara et al., 2006; McNamara and Chen, 2022; Valone, 2024). They predict that animals weight spatial priors heavily in familiar environments. This dependence on spatial priors can produce systematic errors when the environment changes unexpectedly. For instance, an individual entering a familiar room may inadvertently trip over a bag left on the floor, because a learned spatial prior predicts that the floor is unobstructed. When environmental conditions remain stable, spatial priors alleviate cognitive load by reducing the need to direct attention to known object features or locations (Eissa and Kilpatrick, 2023).

In familiar situations, organisms systematically allocate attentional resources toward locations where behaviorally relevant events are most likely to occur (Zuanazzi and Noppeney, 2018, 2019, 2020). This fundamental principle has been extensively studied in a variety of experimental paradigms, most notably the Posner cueing task, which revealed that spatial attention can be manipulated through probabilistic cues that predict target locations (Posner, 1980). While extensive research has explored human attention to expected locations using paradigms like Posner's, direct measurement of spatial attention during navigation has proven challenging in non-human species. Echolocating bats offer a distinct opportunity to bridge this gap, as their active control over sonar signals yields a direct metric of spatial attention (Moss and Surlykke, 2001). Here, we leveraged the directional aim of Egyptian fruit bat echolocation signals to discover that robust spatial priors shift sonar-guided attention away from stimulus changes in familiar environments.

There are more than 1400 species of bats globally, representing approximately 20% of all mammalian diversity (Simmons and Cirranello, 2024). Bats occupy a range of ecological niches, from arid deserts to tropical rainforests, and their diets span fruit, nectar, insects, fish, small vertebrates and even blood. Body sizes among bats are equally diverse, ranging from the bumblebee bat (*Craseonycteris thonglongyai*), among the world's smallest mammals by mass (∼2 g), to flying foxes (*Pteropus*), which can exceed 1 kg in mass and have wingspans over 1.5 m (Monadjem et al., 2020). Egyptian fruit bats (*Rousettus aegyptiacus*) are a medium-sized species of fruit bat (mean mass ∼120 g, forearm length >80 mm, average body length 11–13 cm, wingspan 60 cm) and produce echolocation signals with the tongue (Monadjem et al., 2020). They increase sonar click rate when navigating obstacles, during landing and under low light levels (Danilovich et al., 2015). GPS tagging of foraging Egyptian fruit bats has documented that they fly consistently along stable routes, traveling up to 20 km to foraging sites, and rely on cognitive maps to travel to and from feeding sites (Tsoar et al., 2011; Harten et al., 2020; Toledo et al., 2020; Lourie et al., 2021). This bat species relies on both highly sensitive scotopic vision (Möhres and Kulzer, 1956; El-Mansi et al., 2020) and lingual echolocation (Möhres and Kulzer, 1956; Griffin, 1958; Herbert, 1985; Holland et al., 2005) for orientation. *Rousettus aegyptiacus*' large eyes support relatively high spatial resolution (Heffner et al., 1999) and exhibit low visual detection thresholds indicative of high absolute sensitivity (Danilovich and Yovel, 2019), while retaining some degree of binocular visual field overlap, suggesting the potential for depth perception (Thiele et al., 1991). Vision operates over long distances and can be used to monitor distal landmarks or beacons, while echolocation offers precise range estimation of nearby objects (Danilovich et al., 2015; Danilovich and Yovel, 2019).

Egyptian fruit bats emit and steer echolocation clicks with the tongue (Yovel et al., 2011a,b; Lee et al., 2017). These clicks are produced in pairs and directed off-axis from the midline, allowing the bat to position the maximum slope (‘edge’) of the sonar beam on a target, which benefits localization accuracy (Yovel et al., 2010, 2011a,b; Lee et al., 2017). Importantly, the bats’ directional aim of their sonar serves as an explicit, measurable proxy for moment-to-moment spatial attention, as they direct their sonar, like an acoustic flashlight, towards the objects they inspect or regions in space they expect to find food, shelter or perches (Moss and Surlykke, 2010; Moss et al., 2011). By leveraging the Egyptian fruit bat's active sensing system, we can directly quantify its allocation of spatial attention during navigation and landing across different sensory environments.

In this study, we induced learned spatial priors in Egyptian fruit bats by training them to navigate and land repeatedly on a perch fixed in a stable environment. We then shifted the perch intermittently to test whether spatial priors affected navigation and landing performance. The relative weighting of priors and sensory information was investigated by comparing navigation and landing performance in light and dark environments. We hypothesized that the relative weighting of prior knowledge and real-time stimulus information depends jointly on environmental conditions, spatial attention and the bat's 3D sensory volume. We therefore predicted that bats would exhibit navigation and landing failures when they encountered unexpected changes in familiar settings, particularly in the dark, where sensory volumes are limited to those conferred through echolocation.

## MATERIALS AND METHODS

### Research subjects and experimental setup

#### Subjects

Eight Egyptian fruit bats, *Rousettus aegyptiacus* (Geoffroy 1810), served as subjects in this study and were divided into two groups. The first group consisted of four bats (two males, two females) trained initially in the light (28 lx). One female bat died from natural causes before data collection was completed. Of the four bats trained in the light, all met testing success criteria of 80% successful landings. A successful landing was defined by the bat's physical contact with the perch, including gripping with its feet, in either the first or subsequent approaches to the perch. A failed landing was defined by the bat's absence of contact with the perch on the first or subsequent approaches to the perch. Another group consisted of four bats (two males and two females), initially trained in the dark (0.76 lx). None of the bats initially trained in the dark reached the testing criteria, despite training for multiple days, and therefore only the number of trials run for each of these subjects is reported. Information on the training data collected for all bats can be seen in Table S1. Bat 08 was trained for only 1 day because it struggled to navigate in the room. Bat 06 successfully landed on the perch a few times but lacked consistent performance to progress to the testing phase. The bats' struggle to find the perch in the dark prompted the need to train them initially in the light (Table S1).

#### Ethics statement

The handling and treatment of bats adhered to the guidelines outlined by the American Society of Mammalogists (National Research Council, 2011) and were done in full compliance with the Institutional Animal Care and Use Committee at Johns Hopkins University (protocol number BA23A45). These protocols strictly conformed to the regulations set forth by the Animal Welfare Act and the Public Health Service Policy. The university maintains accreditation by the Association for the Assessment and Accreditation of Laboratory Animal Care International.

#### Experimental setup

**Fig. 1.**
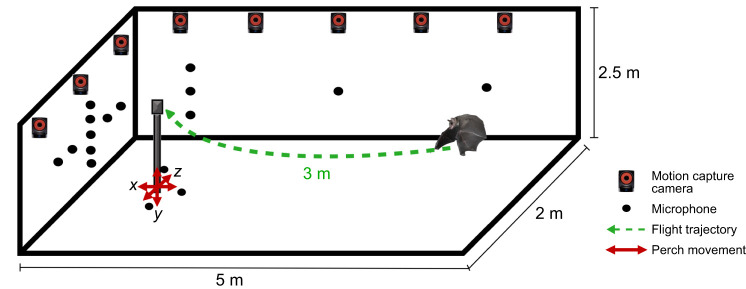
**Schematic diagram of the test room and navigation task.** Schematic diagram of the flight arena showing an example flight trajectory of a bat flying to the perch after being released from the back of the arena. Arrows represent the direction of perch movement (up, down, left, right, front and back) during testing. A 25-channel microphone array was used to record the directional sonar behavior of the bats (not all microphones are shown). Thirteen infrared motion capture cameras were used to track the location of the bat as it flew in the room (not all motion cameras are shown).

Bats were trained to land on a perch (height: 125 cm) in a set location in the room that was a rest area where food was offered. The flight arena, 5.0 m×2.0 m×2.5 m, was lined with acoustic foam on the walls and ceiling, carpeting on the floor and felt placed over reflective objects to mitigate echoes (Fig. 1). In the first phase of training, bats were habituated to the flight room by allowing them to fly freely and naturally find the perch. To control for olfactory cues, food was distributed around the room, but only food on the perch was accessible. Once the bats were accustomed to the flight room and had successfully located the food on the perch, they were released from random locations within the room. A 3 min interval followed each successful landing to allow the bats the opportunity to rest. The testing phase commenced once the bats reached a success criterion of 80% successful landings on the perch for two consecutive days.

Training in the light and dark conditions followed the same procedures; however, only bats trained in the light reliably met the success criterion of 80% successful landings. The bats trained in the dark were excluded from the experiment, because they failed to reach the 80% landing success criterion. Data reported here for tests in the dark are from bats initially trained and tested in the light.

### Experimental procedure and design

The experiment was designed to evaluate bat navigation behavior and sonar-guided attention in a standard condition, where the perch was at a fixed location, and in a probe condition, where the location of the perch was moved. During the experiment, bats navigated to a perch location from one of three release locations at the back of the room: left – 1 m from the back wall, 50 cm to the left of the center of the room; middle – at the back wall, in the center of the room; right – 50 cm from the back wall, 50 cm to the right of the center of the room. Within an experimental test day, each bat was run in 6–8 standard trials, where the perch remained in its original familiar location. Only once per test day was the perch moved to a different location in the room, and this trial is referred to as a ‘perch-moved’ trial. The order of the perch-moved trial within the sequence of trials each day was determined following a pseudorandom schedule, with the constraint that the perch-moved trial was never the first or last trial of a test day. The trial immediately after the perch-moved trial, in which the perch was returned to the standard location, was termed the ‘post-perch-moved’ trial. We intentionally limited the number of perch-moved trials run per bat, to minimize the bats learning about perch location changes. The experiment followed a block schedule design, with each block consisting of 4 days. On 3 out of the 4 days, the perch was moved, averaging three movements per 21 trials. Between blocks, there were 1–2 days of trials where the perch remained stationary.

### Perch-moved conditions

In the perch-moved trials, the perch was placed in a new position in one of the following directions – left, right, front, back, up or down – by either 15 cm (about one body length of a male Egyptian fruit bat) or 30 cm (about two body lengths of a male Egyptian fruit bat) (Fig. 1). This variation was designed to prevent the bat from predicting the moved perch's spatial location. To further prevent the bat from predicting perch movements, the same distance and direction were never repeated within the same block. Occasional equipment failures necessitated the retesting of some conditions and trials. This, along with a deliberately randomized testing schedule and unforeseen events, such as a pregnancy in one of the bats, contributed to variation in the number of trials run with individual bats across conditions. The total number of trials run across conditions for each bat is shown in  Table S2. The number of perch-moved conditions was deliberately limited compared with the standard trials to ensure that the standard position served as the statistical reference point or spatial prior.

### Testing in the dark

Three bats were run in a combined total of 263 trials in the light, and then were retrained in the dark until they met an 80% landing success criterion. They were then run in an additional 131 trials in the dark. This phase of the experiment aimed to determine whether bats relied more heavily on spatial priors when using echolocation alone. It also assessed the Egyptian fruit bat's ability to navigate in the dark after learning the task and the environment in the light. In the dark, the perch-moved trials were confined to 15 cm, following the same experimental procedures as outlined above. Restricting perch-moved trials to 15 cm was based on preliminary findings that bats struggled to land on a perch that was moved by even this distance in the dark.

### Audio and video recordings

A 25-channel microphone array of electret condenser microphones (Pettersson Elektronik, Uppsala, Sweden) was used for acoustic data collection (Fig. 1). Each microphone was calibrated for sensitivity and directionality using a precision microphone (40DP 1/8 inch, G.R.A.S., Holte, Denmark). Echolocation signals picked up by the microphones were bandpass filtered between 10 kHz and 100 kHz at 10× gain (SBPBP-S1, Alligator Technology, Costa Mesa, CA, USA) and digitized at a sampling frequency of 250 kHz. Three markers placed on microphones were used to record the positions and directions of the microphones in the room. The beam patterns of each sonar click were reconstructed using MATLAB v.9.13.0 (R2022b) (The MathWorks Inc., Natick, MA, USA) (Lee et al., 2017), which interpolated the energy spectral density (ESD) of the clicks across all microphones. Corrections for atmospheric absorption and spherical spreading loss were made using recorded temperature and humidity for each day. Beam aim measurements provided a metric for the animal's sonar-guided attention.

In each trial, the bat's 3D flight trajectory was reconstructed using a 13-camera motion capture system (Vicon T40 and T40S, Oxford, UK) operated at a rate of 100 frames s^−1^. Three reflective markers were positioned on a triangle affixed to the bat's head, which were used to track the bat's flight trajectory (Lee et al., 2017). In addition, infrared high-resolution video cameras (Phantom Miro 310, Vision Research, Wayne, NJ, USA), operating at a frame rate of 200 frames s^−1^, confirmed landing behavior.

### Analysis

#### Flight patterns

Bat flight trajectories were assigned to one of four outcomes that cataloged the bat’s flight path during each trial: (1) direct success: the bat flew directly to the perch and landed on it after release; (2) direct failure: the bat approached the perch only once but did not successfully land on it; (3) back-and-forth success: the bat did not land on the perch on its initial approach but successfully landed after executing more than one approach; and (4) back-and-forth failure: the bat approached the perch more than once during its flight but failed to land on it.

#### Sonar beam aim: metric of sonar guided attention

**Fig. 2.**
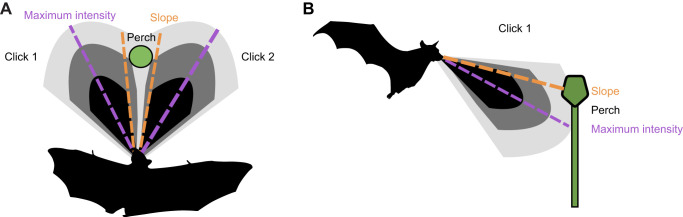
**Sonar beam measurements relative to target.** (A) Left and right horizontal sonar clicks (top view). Click 1 was emitted first, click 2 was emitted second. (B) Vertical distribution of sonar clicks (side view). Egyptian fruit bats direct the region of maximum vertical derivative (the slope) of their sonar beam below the target, rather than pointing the beam's peak at the target. In both A and B, the slope is defined by a −3 dB drop in sound intensity to half the peak value of the signal. This is used to define the shape and width of the sonar beam. Placing the slope at objects instead of the center of the beam increases sensitivity to changes in the target's elevation. Figure inspired by Yovel et al. (2011a,b) and Lee et al. (2017).

The directional aim of the bat's echolocation clicks was used as a metric for the bat's sonar-guided attention. The directional aim of the bat's sonar was quantified in relation to the original and moved perch locations. Egyptian fruit bats produce sonar clicks in pairs, typically placing the maximum slope of each click in the pair at the location of a landing perch (Fig. 2) (Yovel et al., 2010). Clicks within each pair tended to be directed at the same location in the vertical plane in this dataset. In trials where the perch was moved horizontally (i.e. left or right), the direction of the bat's sonar beam aim was determined by the midpoint of each interclick pair on the horizontal plane (Fig. 2A) (Yovel et al., 2011a,b). In trials where the perch was moved vertically (i.e. up or down), the beam aim along the vertical plane was determined by the point where the rate of change of energy with height (energy-to-elevation derivative) was nearly at its peak for each click in the pair, which is just above the highest intensity region of the sonar beam (Fig. 2B) (Eitan et al., 2019). The location with the smaller angular distance to the direction of focus was determined to be the point where the bat's attention was centered, between the standard location and moved perch location. Any sonar signals directed outside a ±10 deg tolerance of the moved or standard perch locations were categorized as undefined. Details of sonar beam analysis are reported in Lee et al. (2017), which provides a framework for this analysis. Additionally, only trials that contained at least 4 clicks were included for analysis. Some clicks were excluded from analysis because of low signal to noise ratio (SNR) or because there was only one click in the click pair.

#### Click rate

In each trial, the number of clicks emitted by the bat during its initial and subsequent approaches to the perch, as well as the flight times (seconds), travel distances (meters) and the Euclidean distance between the bat and the perch were extracted and plotted in MATLAB v.9.13.0 (R2022b). The click rate over time and distance – defined as the number of clicks emitted divided by time (in seconds) and distance (in meters) – for each approach to the perch within a trial was calculated. There was no statistical difference in click rates calculated over time and distance to the perch so for prosperity purposes only click rate calculated over distance is presented. Approach 1 was characterized as the period from the bat's takeoff until it either landed on the perch or passed it during flight. The conclusion of an approach phase was defined by the bat landing on the wall or when the distance from the bat to the perch increased over at least 10 consecutive frames (10 ms). The onset of Approach 2 was determined by a subsequent decrease in the distance to the perch following Approach 1.

Notably, Egyptian fruit bats tend to increase their click rate in the dark or when preparing to land (Yovel et al., 2011a,b; Danilovich et al., 2015). To assess the bats’ click rate as they approached or closely inspected the perch, we computed the Euclidean distance between the bat and the perch for each emitted click pair. The bats' average distance from the perch at which they increased click rate was then extracted. The average click rate at this distance was computed across all approaches to the perch, when the perch was in the standard location, and when it was moved both 15 cm and 30 cm, in the light and dark.

### Statistical analysis

All statistical analyses were conducted using R Studio and SPSS. Because of repeated measures and the nested design of the data, mixed models were used to identify significant relationships. Bat ID was included as a random effect in all mixed models to account for repeated measures; α=0.05 and *P*-values were adjusted for multiple comparisons. Predictors considered across models were: environment (light versus dark), perch displacement [standard; perch-moved 15 cm; perch-moved 30 cm (light only); post-perch-moved (return to standard)], perch displacement direction (left, right, up, down, front, back), and release position (left, middle, right) trial order per day. Landing outcome (landed versus did not land) was used as a predictor in two models (click rate and sonar aim) but was not used when landing itself was the response. Continuous covariates (approach number within trial, performance drift across sessions) were used in exploratory models and excluded from final models when non-significant. Bonferroni's corrections were used for multiple comparisons.

#### Flight patterns

A multinomial generalized mixed model (GLMM) with repeated measures was used to evaluate fixed effects of lighting condition, perch displacement and their interaction on flight patterns, which had four possible outcomes (multinomial): direct success, back-and-forth success, direct failure and back-and-forth failure. *Post hoc* pairwise comparisons were done with estimated marginal means (EMMs).

#### Landing success

Landing success (response outcome: landed versus did not land) was modeled against fixed effects such as perch displacement, lighting condition and release position. A GLMM was used with a binary logistic regression to account for repeated measures, as each bat was subjected to multiple trials across different conditions. We obtained pairwise contrasts of EMMs with Tukey unequal-N adjustment.

#### Sonar beam aim

For each trial, we tallied click pairs aimed at the standard location, the perch-moved location or undefined (>±10 deg of either perch). Because counts were non-normal and often zero-inflated, we used a rank-based approach for the main dataset: a Kruskal–Wallis test across perch displacements (standard; moved 15 cm light; moved 30 cm light; moved 15 cm dark), followed by Dunn's *post hoc* test with a Benjamini–Hochberg correction to identify specific pairwise differences when significant results were observed.

#### Click-rate (calculated over distance and near the perch)

GLMMs were used to model the relationship between click rates – over the approach to the perch and near the perch – across lighting conditions, perch displacements and landing success. Separate GLMMs were fitted for each click-rate variable, with lighting condition, perch displacement and landing success and their interactions included as fixed effects, and individual bat as a random effect to account for individual variability. *Post hoc* pairwise comparisons were performed using EMMs to identify specific differences between levels of lighting conditions, perch displacement and landing success. Pairwise comparisons were made between bats that landed and those that did not land across different lighting and perch displacement conditions. Type III ANOVA were used to evaluate the significance of each factor and interaction in the models. Additionally, a separate Friedman ANOVA followed by a Wilcoxon signed rank test was used to test the difference in click rates within each bat navigating in the dark and light control trials (no perch movement), where the bats reached an 80% success criterion, across all approaches to the perch. Bonferroni corrections were applied to control for multiple comparisons.

## RESULTS

Eight bats participated in this navigation study (four trained in the light, four trained in the dark). The four bats trained in the light reached successful landing criteria of 80% (two males, two females). One female bat died of natural causes before a complete dataset could be collected. The four bats trained in the dark failed to reach the 80% success landing criteria after a combined total of 416 trials. Bats initially trained in the light quickly reached testing criteria again in the dark (Table S1). In total, 394 trials were conducted in the light and dark, across the three surviving bats first trained in the light, including 50 probe trials where the perch was moved (Table S2).

### Flight patterns

**Fig. 3.**
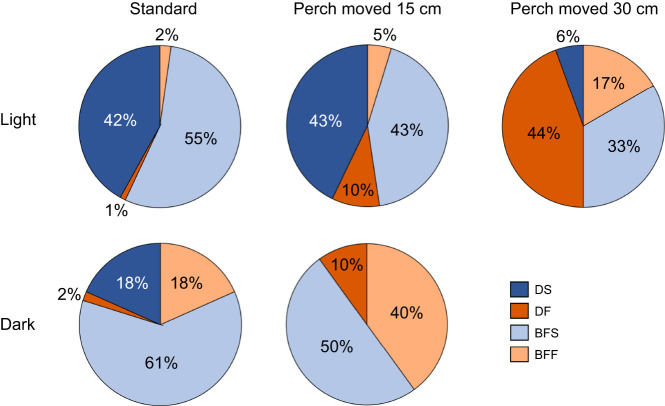
**Flight patterns across different perch displacements and lighting.** Egyptian fruit bats' flight approach and landing outcomes under different perch displacements (standard, perch moved 15 cm and perch moved 30 cm) and lighting conditions (light and dark). Approach and landing performance are categorized into four distinct outcomes: direct success (DS), direct failure (DF), back-and-forth success (BFS) and back-and-forth failure (BFF). Pie charts aggregate a total of 394 trials collected from three individual bats: 263 trials in the light, 179 in light standard, 23 in light moved 15 cm, 18 in light moved 30 cm; 131 trials in the dark, 109 in dark standard and 12 in dark moved 15 cm. Group differences were evaluated using the mixed-effects models described in Materials and Methods; tests were two-tailed.

**Fig. 4.**
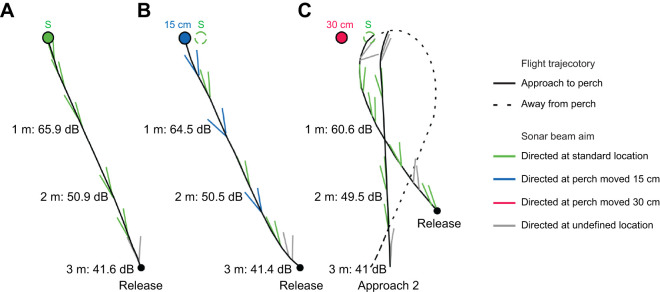
**Schematic diagram of example flight trajectories and sonar beam aim.** Example flight trajectories and directional aim of sonar clicks when the perch was positioned in three different locations in the light: (A) standard location (S), (B) perch moved 15 cm and (C) perch moved 30 cm. Approaches to the perch are illustrated by solid black lines, while departures are shown by dashed black lines. Echolocation clicks are represented by lines along the flight trajectory, whereby color indicates where the click pairs are directed: green, standard perch location; blue, perch moved 15 cm; red, perch moved 30 cm; and gray, undefined location outside the perch locations. Received perch echo levels (dB SPL) were calculated at distances of 1, 2 and 3 m (Tables S4 and S5). Note that echolocation click pairs were sometimes aimed at the standard location when the perch was moved from that location (dashed circles) in the perch moved 15 cm trial and consistently in the perch moved 30 cm trial. Notably, in this example, the bat produced no sonar clicks directed at the perch when it was moved by 30 cm from the standard location; hence, there are no sonar beam clicks denoted in red in C.

**Fig. 5.**
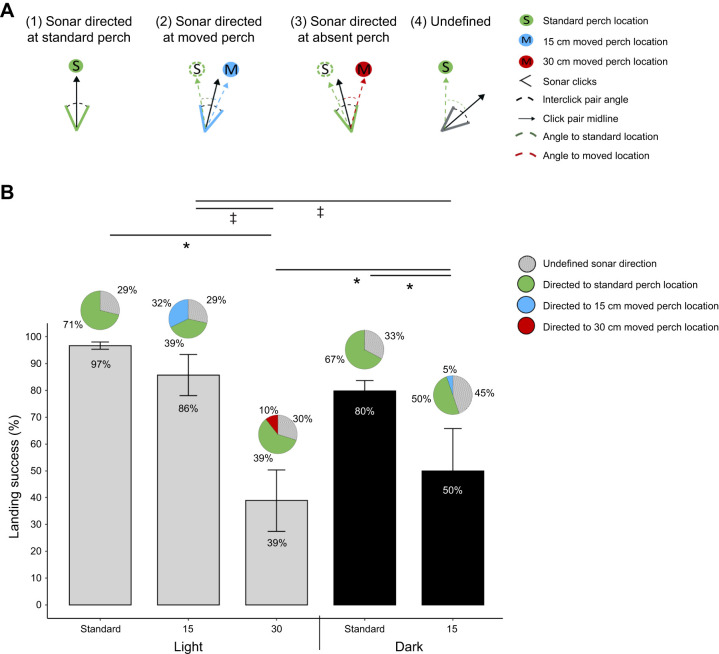
**Bat performance in the light and dark under each condition.** (A) Metric of sonar-guided attention: the directional aim of echolocation clicks. Schematic diagram of sonar click pairs with angular offsets relative to the perch in different conditions. The midline of a click pair (black arrow) in condition 1 is directed towards the standard perch location (S, shown in green). The midline of a click pair in condition 2 is directed towards the 15 cm moved perch location (M, shown in blue). The midline of the click pair in condition 3 is directed towards the standard perch location (S) in the 30 cm perch-moved trial. The midline of a click pair directed outside the two locations of interest in condition 4 was categorized as undefined, based on a ±10 deg tolerance to the nearest perch location (shown in gray). (B) Landing behavior and sonar beam aim reveal the influence of spatial priors on attention and behavior in perch-moved trials. Bar graph of the landing success (means±s.e.m) of bats when the perch was at the standard location, moved 15 cm and moved 30 cm in the light and dark. Pie charts depicted above performance plots for each condition show the aim of the sonar click pair towards the standard perch location (green), the perch moved by 15 cm (blue) or 30 cm (red), or in an undefined direction (gray striped) within that condition. Note when the bat directed its sonar beam toward the standard perch location after the perch was moved by 15 or 30 cm (shown in green), it did so to a location where the perch was absent. Both direct and back-and-forth flights that concluded with perch contact constituted a successful landing. Landing data are from a total of 394 trials collected from three individual bats: 263 trials in the light, 179 in light standard, 23 in light moved 15 cm, 18 in light moved 30 cm; 131 trials in the dark, 109 in dark standard and 12 in dark moved 15 cm. Group differences were evaluated using the binomial mixed-effects models and Tukey unequal-*N* procedure described in Materials and Methods. Asterisks denote significant differences between landing success across conditions (*P<0.05). The pie charts summarize a total of 393 click pairs over 69 trials from three bats. Trials were classified as standard, moved or undefined: light standard (*n*=30), light moved 15 cm (*n*=5), light moved 30 cm (*n*=8), dark standard (*n*=18), dark moved 15 cm (*n*=8). Beam-aim click pairs were compared with a Kruskal–Wallis test followed by Dunn's *post hoc* contrasts (Benjamini–Hochberg correction). Significant differences between sonar beam-directing behavior across conditions are denoted by double-daggers (^‡^*P*<0.05). All tests were two-tailed.

Bats’ flight patterns include direct successes (DS), back-and-forth failures (BFF), back-and-forth successes (BFS) and direct failures (DF) (Fig. 3). Both DS and BFS constituted a successful landing. Example flight patterns are shown for different perch displacements in the light in Fig. 4. A multinomial logistic regression revealed significant effects of lighting and perch location on flight patterns, with several key contrasts showing differences in flight patterns (GLMM; χ_35,343_=175). The model converged with a final deviance of 316.82 and an Akaike information criterion (AIC) of 693.64. Bats showed similar flight patterns to the standard perch location and to the perch moved 15 cm in the light (*P*>0.05 for DS, BFF, BFS and DF). When the perch was displaced by 30 cm in the light, bats made fewer direct successful landings compared with both the standard (*P*<0.001) and 15 cm perch-moved conditions (*P*=0.048). Bats failed to land on the perch most often when it was moved by 30 cm in the light compared with when it was moved by 15 cm in the light and dark (*P*<0.05). In standard trials, bats in the dark made more repeated back-and-forth approaches to the perch before successfully landing than in the light (*P*<0.01). In contrast to the standard location in the dark, bats showed no direct landings after the perch was moved by 15 cm (*P*<0.001). Bat performance in the light and dark 15 cm perch-moved conditions was also significantly different (*P*<0.001) (Fig. 5). There were no significant differences in flight patterns among individual bats (*P*>0.05 for all bats), although bat 2 tended to exhibit more BFS landings compared with DS landings across all conditions (Fig. S1).

### Sonar click rate

Bats tend to increase their click rate as they approach a landing perch (Yovel et al., 2011a,b). Sonar click rate is therefore reported (a) for the entire approach to the perch and (b) as the bat neared the perch (<1.5 m from the perch). Figs S2 and S3 show sonar click rate across different perch displacement magnitudes (standard versus perch moved 15 cm versus perch moved 30 cm), lighting conditions (light versus dark) and landing conditions (landed versus did not land). There were no significant differences in click rates across different perch displacements (GLMM: *F*_3,160_=0.127, *N*=176, *P*=0.944; GLMM: *F*_3,160_=1.954, *N*=176, *P*=0.123) (Fig. S2). Lighting was a significant factor in predicting click rate (GLMM: *F*_1,160_=16.421, *N*=176, *P*<0.01; GLMM: *F*_1,160_=10.583, *N*=176, *P*<0.01) (Fig. S3). Landing was only a significant predictor of click rate near the perch (GLMM: *F*_1,160_=13.814, *N*=176, *P*<0.001) but not over the entire approach to the perch (*F*_1,160_=2.024, *N*=176, *P*=0.157). There were no significant interactions between perch displacement, lighting and landing (GLMM: *F*_2,160_=0.921, *N*=176, *P*=0.400; GLMM: *F*_2,160_=0.709, *N*=176, *P*=0.494). In standard trials, most bats produced statistically higher click rates in the dark than in the light over the entire distance to the perch [Friedman ANOVA: χ^2^_2_=114.920, *N*=88, *N*=3, *P*<0.001; bat 01 Wilcoxon signed rank test: *z*=2.203, *N*=13, *P*=0.028; bat 02: *z*=2.982, *N*=12, *P*<0.01; and bat 03: *z*=3.622, *N*=17, *P*<0.01] (Bonferroni, *m*=3, *P*_adj_=0.012).

### Landing success

The bats' landing success was significantly influenced by both lighting conditions and perch movement. When the perch was moved, the bats' sonar beam-directing behavior revealed that they continued to direct their spatial attention to the standard perch location (where the perch was absent), which corresponded with failure to find the perch at its new position (Movie 1). GLMM analysis showed that lighting condition (*P*<0.01) and perch displacement (*P*<0.01) were significant predictors of landing success, with the model explaining 88.9% of the variance (GLMM: *F*_36,328_=0.736). The analysis showed no significant relationship between landing success and perch displacement direction (left, right, up, down, front, back) (*P*=0.324), landing success over time (*P*=0.920), release location (left, middle, right) (*P*=0.888), individual bat (*P*=0.093), or interactions between these variables (unequal-*N P*>0.05 for all interactions). Therefore, data for different perch movement directions and release locations were combined, as these factors did not significantly affect landing success. The bats exhibited comparable landing success when the perch was at the standard location in trials both preceding (97%) and following (90%) the perch moved trials across all trials (*P*>0.05 for both test displacements) (Table S2). In the dark, landing success was lower in the trials following perch movement (60%) compared with the trials preceding them (80%), however, because of the large variation, this was not statistically significant (*P*>0.05).

In the light, bats landed successfully on the perch in 86% of the trials after it was moved 15 cm, a landing success rate that was not significantly different from that with standard perch location (*P*=0.810). In contrast, bats landed successfully in only 39% of the trials when the perch was moved by 30 cm in the light. This decrease in landing success after the perch was moved 30 cm was statistically significant compared with both standard (*P*<0.01) and perch-moved 15 cm conditions (*P*<0.01) (Fig. 5). In the dark, bats showed a decrease in landing success to 50% when the perch was moved by 15 cm, compared with 80% when it was in the standard location (GLMM: *F*_36_,_328_=0.736; unequal-*N P*<0.01). The bat's landing performance was significantly lower when the perch was moved by 15 cm in the dark (30% reduction from standard location) (*P*=0.012) (Fig. 2).

### Sonar beam direction

The bats' sonar beam-directing behavior varied with the magnitude of perch displacement (15 cm versus 30 cm) and lighting conditions, suggesting differences in their ability to locate/inspect the perch under different perch-moved conditions. Larger perch displacements (30 cm) and dark conditions led to fewer clicks directed at the moved perch location with more failures to land. There were significant differences in the number of echolocation click pairs directed at the new perch location (Kruskal–Wallis χ^2^=6.714, d.f.=2, *P*=0.035) and a non-significant trend in the number of echolocation click pairs directed at undefined locations (Kruskal–Wallis χ^2^=5.48, d.f.=2, *P*=0.064) in the perch-moved trials. There were no significant differences in the number of click pairs directed at the standard perch location (Kruskal–Wallis χ^2^=1.8422, d.f.=2, *P*=0.398) in the perch-moved trials. Even when the perch was moved from the standard location, bats continued to aim their sonar beam to this region of the room, directing 39% of their clicks in the light and 50% of their clicks in the dark at the standard location in perch-moved trials (Fig. 5; Table S3).

In the light, when the perch was moved 15 cm, bats directed clicks toward the moved perch location and the standard perch location, dividing their sonar attention between the standard and displaced perch positions (Fig. 5; Table S3). They directed 32% of clicks toward the moved perch location and 39% toward the standard location (Fig. 5; Table S3). This behavior differed significantly from the perch moved 30 cm in the light condition, where bats directed only 10% of clicks toward the moved perch location (Dunn's *post hoc* test: *z*=−2.213, *P*=0.0202). It also differed significantly from the perch moved 15 cm in the dark condition, where bats directed only 5% of clicks toward the moved perch location (Dunn's *post hoc* test: *z*=−2.410, *P*=0.0239; Fig. 5; Table S3). There were no significant differences in the number of clicks directed toward the moved perch location between the perch moved 30 cm in the light and perch moved 15 cm in the dark conditions (Dunn's *post hoc* test: *z*=0.478, *P*=0.3162). In both these conditions, bats failed to inspect the perch at the new location and instead directed the sonar beam towards the standard location (where the perch was previously located), resulting in fewer successful landings on the perch (Fig. 5; Table S3).

Across standard trials, clicks where the beam was not directed at one of the perch locations, termed ‘undefined’, represented 29% (in the light) and 33% (in the dark) of the total clicks. When the perch was moved by 15 cm in the dark, bats directed a larger percentage (45%) of clicks at undefined locations in the room, compared with 30% in the perch-moved 30 cm in the light condition (Dunn's *post hoc* test: *z*=0.011, *P*=0.0333).

## DISCUSSION

Our study exploited the Egyptian fruit bat's dual sensing systems, echolocation and vision, to probe the relative weighting of sensory information and spatial priors in guiding navigation and landing. The bat's landing performance and sonar inspection behavior were differentially impacted by available sensory cues. When bats had access to both vision and echolocation in the light, the perceptual load could be shared across the two sensory systems, with vision providing a longer operating range than echolocation (Danilovich and Yovel, 2019). Bats initially trained in the dark failed to find the perch at a fixed location over hundreds of trials (see Table S1). This contrasts with the group of animals first tested in the light and later retested in the dark. In the group retested in the dark, bats landed on the perch displaced by 15 cm in only 50% of trials and directed their sonar to this new perch location only in 5% of the trials. The difference between navigation performance in perch-moved conditions in the light and dark suggests that the combination of vision and echolocation yields a larger sensory volume than echolocation alone. Sensory volume, in turn, appears to interact with the relative weighting of spatial priors. When bats had access to echolocation alone in the dark, they showed a heavier reliance on spatial priors than in the light. In both light and dark conditions, when animals failed to find the relocated perch, they directed their sonar to its expected location.

### The role of spatial priors in navigation

Spatial priors refer to knowledge or expectations about the location of objects or events in the environment based on experience or learned information (Richards et al., 2020; Beitner et al., 2021; Zhang et al., 2022). For example, when navigating a familiar environment, e.g. walking from the living room to kitchen of one's home, an individual may rely on spatial priors to anticipate where furniture, doors or obstacles are located. In familiar settings, bats navigate complex three-dimensional environments by relying on learned spatial information, reducing the need for continuous sensory inspection and instead executing pre-planned movements based on expected encounters in the environment (Griffin, 1958; Neuweiler and Möhres, 1967; Jensen et al., 2005; Winter and Stich, 2005). If the environment is stable, priors greatly reduce cognitive load. However, when the environment changes unexpectedly, reliance on spatial priors can result in errors (Nassar et al., 2010; Kessler et al., 2024), as the bats exhibited in this study.

Spatial priors were established by training bats to fly to a perch at a fixed location. Expectations were violated on intermittent trials by displacing the perch 15 or 30 cm. In both the light and dark, bats exhibited navigation and landing failures on perch-displaced trials, showing higher weighting of spatial priors when vision was excluded. In the light, where bats had access to both vision and echo information, they successfully landed on the perch in 86% of trials after it was displaced by 15 cm, but only 39% of trials after it was moved by 30 cm from its original position. In most navigation failure trials, bats flew to the original perch location, suggesting that spatial priors interfered with localization of the perch's new position. Of the 39% of trials where bats successfully landed on the perch displaced by 30 cm in the light, only 6% were direct successful landings, with the remaining 33% showing multiple attempts before finally reaching the perch. Among navigation failures in the light, 44% were direct failures and 17% were back-and-forth flights preceding failures, where the bats made multiple attempts but ultimately did not land on the perch. In the dark, bats missed the perch 50% of the time when it was displaced by only 15 cm, and when they did land, none occurred on the first approach to the perch. These behaviors indicate that strong spatial priors drive flight path selection to the perch's original location. Bats made multiple approaches to the original location and only after repeated circling did they find the perch at its new location.

Laboratory and field observations document instances where bats continue to navigate according to learned spatial priors, even when confronted with environmental modifications (Griffin, 1988). For instance, historical accounts by Blatcheley (1896) documented little brown bats, *Myotis lucifugus*, flying into newly placed doors in previously open flyways, often with fatal consequences (Blatcheley, 1896). Neuweiler and Möhres (1967) observed that greater false vampire bats, *Megaderma lyra*, maintained a pose required to squeeze through a previously narrow passage, even after the passage was widened (Neuweiler and Möhres, 1967). Griffin (1958) trained bats to navigate a gap in a wall, and when the gap was relocated, they repeatedly crashed (Griffin, 1958). von Helverson and Winter (2005) observed glossophagine bats repeatedly returning to the previous location of a nectar feeder, despite its relocation (von Helverson and Winter, 2005). Stich and Winter (2006) showed that bats failed in tasks when food sources were offered in new spatial contexts, suggesting a reliance on spatial memory (Stich and Winter, 2006). The authors in many of these studies attributed the bats' difficulty in these tasks to their strong dependence on spatial memory, but they did not measure where the animals directed their sonar attention, leaving a gap in understanding the underlying cognitive and perceptual processes of navigation failures.

### Sonar beam-directing behavior during navigation

The bat's sonar beam aim operates like an acoustic flashlight, revealing the presence of objects only where it is directed (Moss and Surlykke, 2010). Thus, the bat's active control over the directional aim of its sonar beam displays the region of space it is inspecting. In both the light and dark, bats produced directionally aimed sonar clicks, which yielded a metric of their moment-to-moment spatial attention to regions of the environment. When animals failed to find the moved perch, regardless of lighting conditions, they directed their sonar attention to the location of the absent perch. In the light, where bats had access to vision and echolocation, they commonly landed on the perch in the new location after it was displaced by 15 cm but in only 39% of trials following the 30 cm perch displacement. In the 15 cm perch displacement trials in the light, bats alternated the directional aim of their echolocation beam between the new perch location and the old one, suggesting that bats used echolocation to monitor the perch position, even when visual information was available. However, in the 30 cm perch displacement condition, only 10% of sonar clicks were directed at the new landing location. In the dark, after the perch was moved by only 15 cm, only 5% of sonar clicks were directed to the new location. Information about the perch location was well within the estimates of their sonar sensing volume as they approached the perch, but they failed to use echoes to guide their navigation and landing. The mean azimuthal beam width of the Egyptian fruit bat at 35 kHz is 25.2 deg, and the mean elevational beam width is 36.6 deg (Lee et al., 2017). From the release point, all perch displacements fell within 10 deg of the standard location; and using standard spreading loss equations (Stilz and Schnitzler, 2012; Finger et al., 2022), we calculated that bats should have received echoes of the order of 41–41.5 dB SPL at the release location when the landing perch was displaced by 15–30 cm. Therefore, bats should have received audible echoes from the perch at both 15 cm and 30 cm perch displacements to guide navigation and landing in the dark.

### Why do bats fail to find the perch? Priors direct spatial attention and perception

One possible explanation for the navigation and landing errors observed in this study is that priors direct spatial attention and serve to filter sensory perception. Michael Posner's (1980) foundational work on visual attention advanced the idea that attention acts as a spotlight, selectively enhancing the processing of stimuli within its volume while filtering out or suppressing less relevant information outside. This selective allocation of cognitive resources has been shown to improve response times and accuracy for attended stimuli (Summerfield and Egner, 2009; Zhang et al., 2022). In familiar environments, spatial priors guide the spotlight of attention toward areas where obstacles or targets are expected based on experience (Beitner et al., 2021; Zhang et al., 2022).

In the attention spotlight framework, the bat's reliance on spatial priors may have anchored its attentional focus on the expected perch location, limiting its sonar-guided attention and navigation to the displaced perch in some of the trials. With a 15 cm perch displacement, the angular offset was small relative to the standard location (Table S4), falling within both the bat's visual and sonar sensing volumes and its spotlight of attention (inferred from the directional aim of its sonar). When visual information is available, Egyptian fruit bats' field of view has the highest acuity in its area centralis at ∼27 deg (±13–14 deg from the line of gaze) (Heffner et al., 1999). They also have high spatial resolution (Heffner et al., 1999) with low detection thresholds that indicate high absolute sensitivity (Thiele et al., 1991). In the light, bats directed their sonar at both the 15 cm-shifted and original perch locations, suggesting that they detected (Table S4) the displaced perch, which is consistent with their 86% successful landings across trials. Following a 30 cm perch displacement in the light, the bats successfully landed in only 39% trials, even though the perch fell within the sensory volumes of its visual and echolocation systems (Table S4).

Navigation and landing failures following small perch displacements suggest that the Egyptian fruit bat's blunders may stem from misdirected attention. In humans, the consequences of misdirected attention result in failures to detect unexpected stimuli or events, a phenomenon known as inattentional blindness.

### Inattentional blindness

In humans, attentional focus can be driven by task instructions or expectations. This can result in failures to notice unexpected but salient stimuli, a well-documented phenomenon known as inattentional blindness (Simons and Chabris, 1999). For example, there are many reports of walkers, drivers and pilots who fail to notice objects within their visual field of view when engaged in other tasks, such as cell phone conversations (Simons and Chabris, 1999; Strayer and Drews, 2007; Hyman et al., 2010; White and O'Hare, 2022). Similarly, in our study, bats may have been blind to the new perch location, because they directed their attention to an adjacent region where the perch was typically located.

Only two other studies have attempted to study inattentional blindness in animals – horses and dogs – but their findings were inconclusive (Gabor et al., 2019; Cox et al., 2023). In these studies, animals repeatedly navigated down a corridor, with half the subjects receiving a reward at the end and the remaining half receiving no reward. A novel stimulus was presented in some of the trials, and researchers attempted to measure the subject's reaction to the novel stimulus. Notably, the authors presented weak objective metrics for stimulus perception, spatial attention or inattentional blindness. By contrast, the Egyptian fruit bat's sonar beam-directing behavior in the present study yielded a quantifiable metric of spatial attention, and in trials where bats missed the displaced perch, they also failed to direct their sonar to inspect the perch's new location.

If the Egyptian fruit bat's failure to find the displaced perch can be attributed to inattentional blindness, this would suggest that this phenomenon could be an evolutionarily conserved aspect of cognition in mammals. However, given the distant phylogenetic relationship between bats and humans, it is also plausible that these processes evolved independently. Investigating this ambiguity across a broader range of species presents an exciting opportunity to explore whether performance errors reflect homology (shared ancestry) or convergence (independent evolution). The adaptability of our experimental paradigm to diverse species provides a platform to assess this across species of distinct evolutionary lineages. Such comparative investigations can help to identify selective pressures driving the evolution of cognitive processes and determine whether inattentional blindness is a universal trait or specific to certain evolutionary lineages.

### Alternative explanations

It is possible that the Egyptian fruit bats in our task detected the moved perch but nonetheless followed learned spatial priors to guide navigation decisions. In Bayesian models, decisions are made by evaluating new sensory information in the context of prior knowledge (McNamara and Houston, 1980; McNamara et al., 2006; Valone, 2024). The relative weighting of new information can be influenced by sensory availability (i.e. light and dark) and its similarity to the learned spatial prior (standard perch location versus 15 cm displacement, standard versus 30 cm displacement). With a 15 cm perch displacement in the light, the bat allocated the directional aim of its sonar between the original and new perch locations. According to this interpretation, the 15 cm displacement was within a window where the new sensory information was sufficiently similar to the original spatial prior to guide successful navigation and landing. In the dark, the bat's sonar beam-directing behavior was strongly influenced by the spatial prior, potentially due to the limited information carried by sonar echo returns. While it remains possible that the bats detected the displaced perch but decided to fly to the familiar location, sonar beam-directing behavior during failed landings indicates that the displaced perch likely received inadequate attention to enable adaptive landing maneuvers, because the sonar beam remained locked onto the original location.

### Conclusions

Our study demonstrates that spatial priors can interfere with bat navigation and landing in both the light and the dark. We leveraged the directional aim of the Egyptian fruit bat's echolocation signals to monitor its spatial attention to objects in the environment and discovered that spatial priors drive sonar inspection of familiar regions, which in turn compromises the use of updated sensory information. Finally, we offer an answer to a longstanding question about bat navigation failures in familiar settings by demonstrating that encoded spatial expectations can alter an animal's attention/perception and action selection in navigation and landing.

## Supplementary Material

10.1242/jexbio.250551_sup1 Supplementary information
