# Supplementary material for: Spatial priors affect sensory weighting in navigation and landing in Egyptian fruit bats
Source: J Exp Biol. 2025 Sep 24;228(18):jeb250551. doi: 10.1242/jeb.250551 (PMC12517345; doi:10.1242/jeb.250551)
Supplement: Supplementary information [file jexbio-228-250551-s1.pdf]

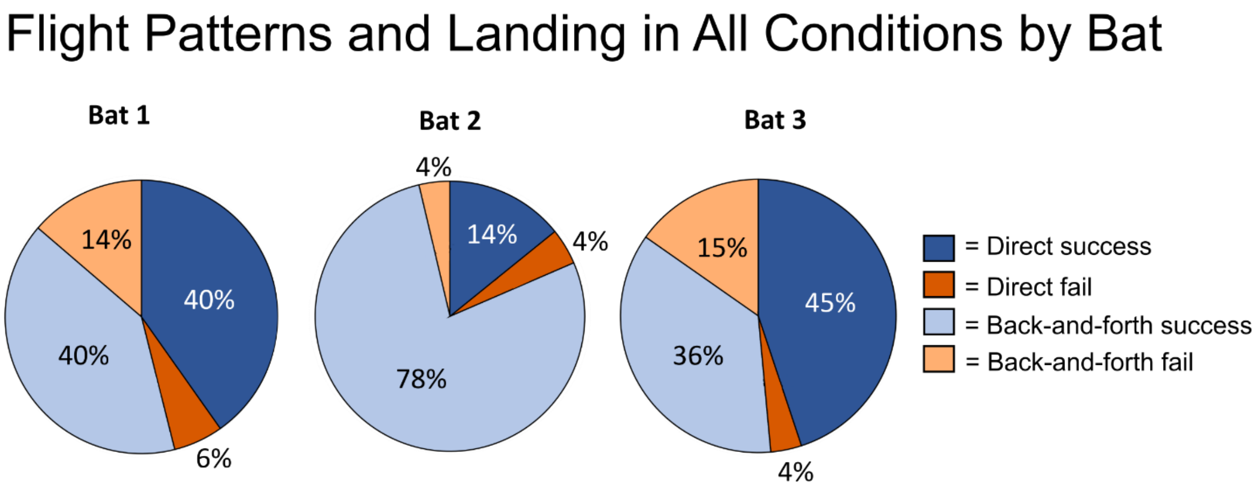

Fig. S1. Flight Patterns and Landing of Individual Bats Across All Conditions. Pie charts show the flight patterns of each bat (Bat 01 (n = 102), Bat 02 (n = 158), Bat 03 (n =134)) across all testing conditions. Bat 2 predominantly exhibited back-and-forth flights, whereas Bats 1 and 3 displayed a more varied distribution of flight patterns.

Journal of Experimental Biology • Supplementary information

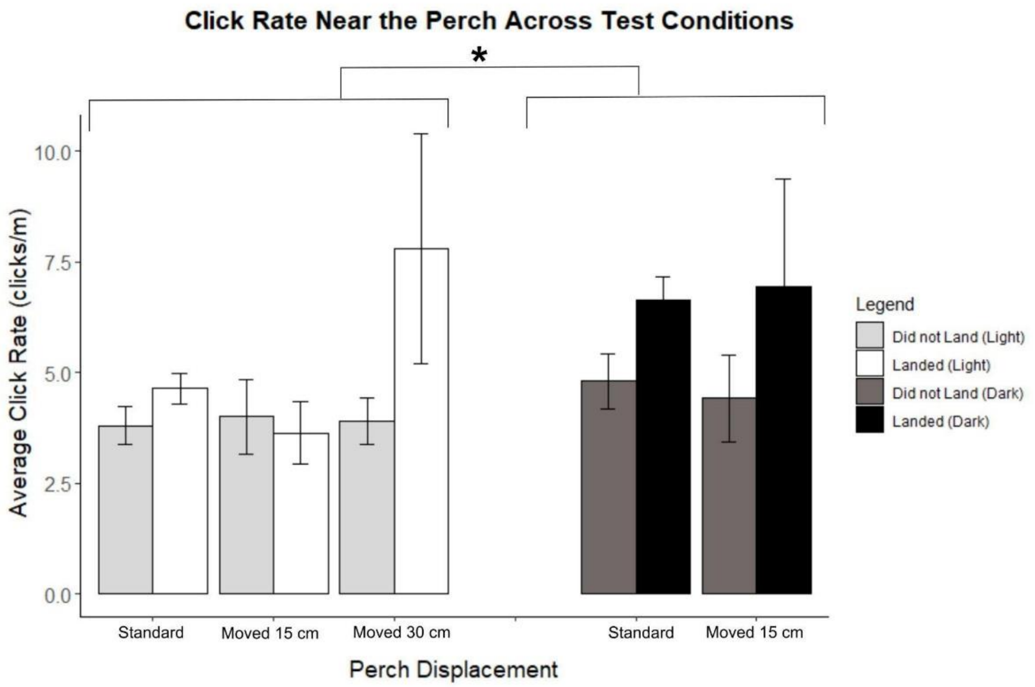

Fig. S2. Echolocation Click Rates Near the Perch. Bar graphs depicting the average echolocation click-rate (±s.e.) of bats within a 1.5-meter radius of the perch when it did and did not land, compared across perch positions in both light and dark conditions. Sonar click-rate did not significantly differ between landing and not landing, though in the 30 cm perch displacement condition and in the dark bats showed higher click rates when they landed. Asterisk indicates significance. Figure aggregates 176 trials from three bats: 123 trials in the light, 85 in light-standard, 18 in light-moved 15cm, 20 in light-moved 30cm; 53 trials in dark, 37 in dark-standard, 16 in dark-moved 15cm. Click rates are reported for all approaches to the perch within a trial. Click rates were analyzed with separate GLMMs as described in the methods; astericks indicate signficance ($p < 0.05$). Two tailed tests were used.

Journal of Experimental Biology • Supplementary information

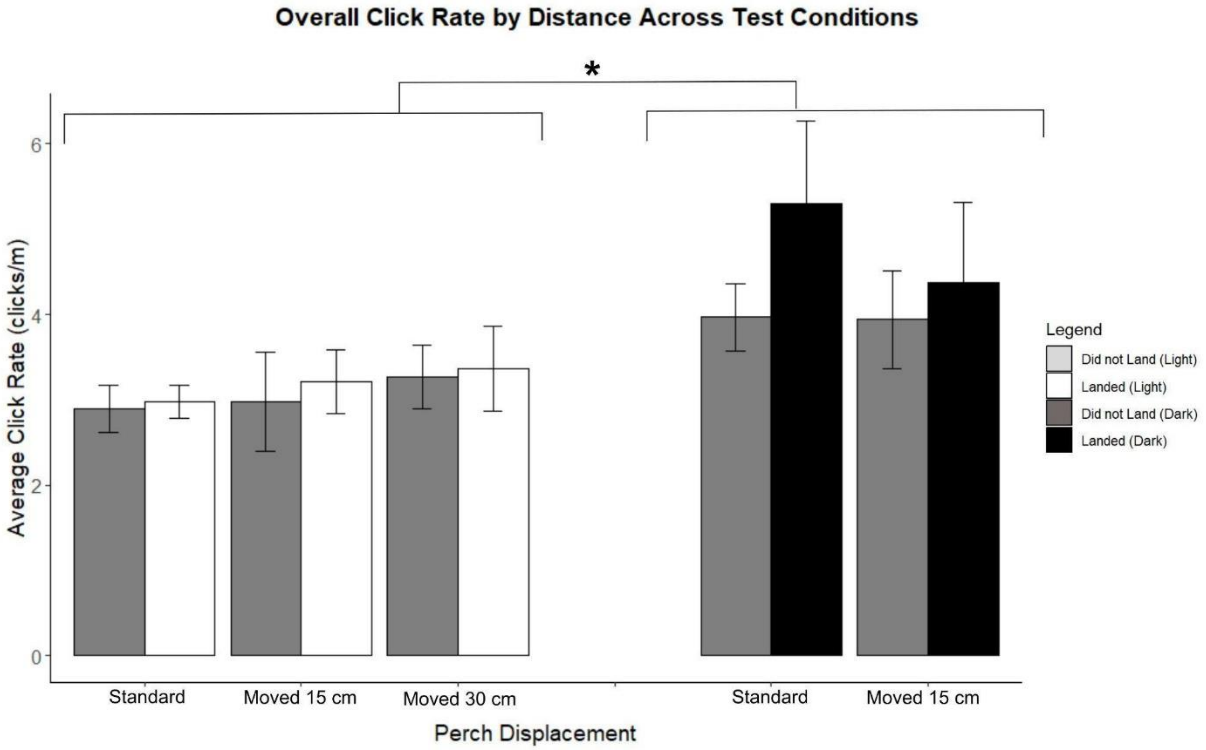

Fig. S3. Echolocation Click Rates Over All Testing Conditions. The average click rate (±s.e.) of bats is shown, separated by light versus dark conditions and whether the bats landed or did not land. Bats produced significantly more sonar clicks in the dark compared to the light. Asterisk indicates significance. The figure aggregates 176 trials from three bats: 123 in the light, 85 in light-standard, 18 in light-moved 15cm, 20 in light-moved 30cm; 53 trials in the dark, 37 in dark-standard, 16 in dark-moved 15cm. Click rates are reported for all approaches to the perch within a trial. Click rates were analyzed with mixed models as described in the methods; astericks indicate signficance ($p < 0.05$). Two tailed tests were used.

Table S1. Summary of Training Data. Left columns show the number of training trials required before individual bats reached criterion performance of 80% successful landing in the light and retraining in the dark. Asterisks show which bats met this criterion. Those that did not meet the criterion yielded no performance data. First landing on the perch is the number of trials recorded before each bat successfully landed on the perch for the first time. On average, bats received 6-8 training trials per day, mirroring the number of trials bats received during testing sessions. Right columns show the number of trials run initially in the dark. No bat reached the 80% successful landing criterion under this condition, and only one bat landed on the perch at all.

		Light (# of trials)				Dark (# of trials)			
Initial Training	**Bat**	**1***	**2***	**3***	**4***	**5**	**6**	**7**	**8**
	**Total Training**	72	9	43	39	39	295	57	12
	**First landing on perch**	17	2	15	13		13		
Retraining in the dark	**Bat**	**1***	**2***	**3***					
	**Total Training**	26	0	32					
	**First landing on perch**	17	0	27					

Table S2. Landing Performance Across Experimental Conditions. Summary of the landing success and failure for each bat under three experimental conditions: standard, perch-moved 15 cm, and perch-moved 30 cm and the trial after the perch was moved. For each condition, the total number of trials and the percentage success (SR%) for each bat are displayed. The data is divided into two conditions: initial testing in light and retesting in the dark. The perch-moved 30 cm condition in the dark was not tested.

		Standard				Perch-moved 15 cm				Perch-moved 30 cm				Post-moved			
	**Bat**	**Land**	**Fail**	**Total**	**SR (%)**	**Land**	**Fail**	**Total**	**SR (%)**	**Land**	**Fail**	**Total**	**SR (%)**	**Land**	**Fail**	**Total**	**SR (%)**
**Light**	1	36	1	37	97	6	0	6	100	1	3	4	25	9	1	10	90
	2	77	2	79	97	8	2	10	80	3	4	7	43	19	0	19	100
	3	60	3	63	95	6	1	7	86	3	4	7	43	11	3	14	79
**Dark**	1	28	9	37	76	2	3	5	40					0	3	3	0
	2	33	2	35	94	3	1	4	75					4	0	4	100
	3	26	11	37	70	2	1	3	67					2	1	3	67

Journal of Experimental Biology • Supplementary information

Table S3. Beam Aim Direction to Perch in Light and Dark Tests. Average number of click pairs directed at the standard location, and new location after the perch was displaced by 15 cm and 30 cm from the standard location. No data were collected for the perch-moved 30 cm condition in the dark. A click pair directed outside the two locations of interest were categorized as undefined, based on a ±10° tolerance to the nearest perch location. This table shows that bats tested in the light tended to direct their sonar beam toward the standard and moved locations after the perch was displaced by 15 cm but predominantly toward the standard location after the perch was displaced by 30 cm. Bats tested in the dark tended to direct their sonar towards the standard location after the perch was displaced by 15 cm.

		Standard (avg. click pairs)			Perch-moved 15 cm (avg. click pairs)			Perch-moved 30 cm (avg. click pairs)		
	**Bat**	**Standard**	**Moved**	**Undefined**	**Standard**	**Moved**	**Undefined**	**Standard**	**Moved**	**Undefined**
**Light**	1	3.6		0.8	2.0	2.5	1.5	3.0	0.3	2.3
	2	2.8		1.7	5.0	0	1.0	3.5	1.0	1.5
	3	4.8		1.9	1.0	2.0	2.0	5.0	0	1.0
**Dark**	1	4.5		3.0	5.0	0	2.3			
	2	2.4		1.9	1.0	2.0	2.0			
	3	6.4		2.0	2.0	1.0	3.0			

Table S4. Calculated Received Echo Levels at Horizontal and Vertical Perch Offsets: Calculated received-echo levels (REL) at 35 kHz from the 15 cm and 30 cm displaced perch for 105 dB sonar clicks directed at the standard perch location. Values are given for three different distances/points in time during the approach to the perch (3 m, 2 m, 1 m). The table lists: on-axis REL, geometric offset, off-axis beam loss, adjusted REL, and the margin above the 25 dB SPL detection threshold (DT) at 35 kHz.

**Azimuth**										
**Distance**	**On-Axis REL (dB)**		**Angle to Perch (°)**	**Loss (dB)**	**Adjusted REL (dB)**		**Angle to Perch (°)**	**Loss (dB)**	**Adjusted REL (dB)**	**Margin above DT (dB)**
3.0 m	41.6	**15 cm Offset**	2.9	0.15	41.4	**30 cm Offset**	5.7	0.62	41	16
2.0 m	50.9		4.3	0.35	50.5		8.5	1.38	49.5	24
1.0 m	65.9		8.5	1.38	64.5		16.7	5.27	60.6	36

**Elevation**										
**Distance**	**On-Axis REL (dB)**		**Angle to Perch (°)**	**Loss (dB)**	**Adjusted REL (dB)**		**Angle to Perch (°)**	**Loss (dB)**	**Adjusted REL (dB)**	**Margin above DT (dB)**
3.0 m	41.6	**15 cm Offset**	2.9	0.08	41.5	**30 cm Offset**	5.7	0.3	41.3	16
2.0 m	50.9		4.3	0.17	50.7		8.5	0.67	50.2	25
1.0 m	65.9		8.5	0.67	65.2		16.7	2.58	63.3	38-40

Journal of Experimental Biology • Supplementary information

Table S5. Parameters and Equations Used in Received Echo-Level Analysis. Acoustic parameters and equations (Stiltz & Schnitzler, 2012; Finger et. al., 2022) used in calculating the detection threshold and received echo levels at a given distance. Supplementary angular attenuation formulas used for the perch moved trials are presented below.

Parameters and Equations Used in the Received Echo-Level Analysis			
**Category**	**Term**	**Value**	**Rationale**
**Source Level**	SL @ 0.1m	105 dB SPL @ 0.1 m	Within the range of source levels observed in this study (~ 95 dB to 115 dB)
**Propagation**	α	1 dB m^{-1}	35 kHz, moderate T/RH (Stilz & Schnitzler 2012)
**Target Strength**	TS	– 40 dB	Large-insect reflector (Stilz & Schnitzler 2012)
**Detection Threshold**	DT	25 dB SPL @ 35 kHz	Behavioral hearing threshold (Koay, Heffner & Heffner 1998)
**Beam pattern**	Azimuth –3 dB half-width	12.6°	Mean Azimuth beamwidth 25.2° (Lee et al. 2017)
	Elevation –3 dB half-width	18°	Mean Elevation beamwidth 36.6° (Lee et al. 2017)
**Geometry**	Δx	0.15 m, 0.30 m	Horizontal / vertical test displacements
	R	3 m, 2 m, 1 m	Distances used in echo-level analysis
**Formulas**	Detection threshold equation	Detection threshold = Source level - Transmission loss + Target strength	Stilz and Schnitzler (2012)
	Offset angle	θ=arctan(Δx/R)	
	Azimuthal loss	$\Delta L_{azi} = 3(\theta/12.6^{\circ})^2$ dB	Quadratic fit giving –3 dB at 12.6°
	Elevational loss	$\Delta L_{ele} = 3(\theta/18^{\circ})^2$dB	Quadratic fit giving –3 dB at 18°

Journal of Experimental Biology • Supplementary information

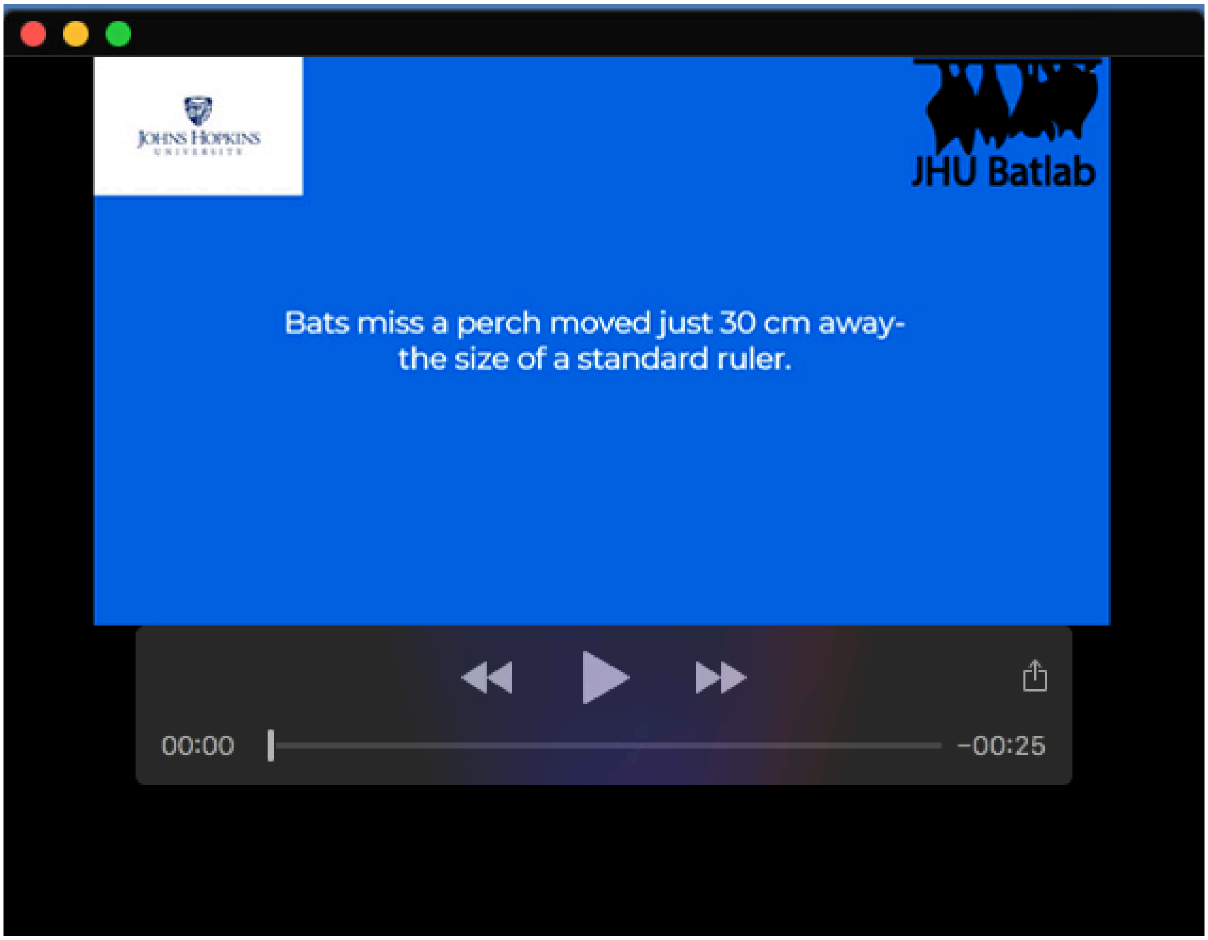

Movie 1. Spatial Priors Direct Sonar Guided Attention. Example flight trajectories and directional aim of sonar clicks when the perch was in the original and moved 30 cm positions in the light. Approaches to the perch are illustrated by solid black lines, echolocation clicks are represented by blue lines along the flight trajectory, and colored circles represent perch locations.
